# Single trial Bayesian inference by population vector readout in the barn owl’s sound localization system

**DOI:** 10.1371/journal.pone.0303843

**Published:** 2024-05-21

**Authors:** Brian J. Fischer, Keanu Shadron, Roland Ferger, José L. Peña

**Affiliations:** 1 Department of Mathematics, Seattle University, Seattle, Washington, United States of America; 2 Dominick P Purpura Department of Neuroscience, Albert Einstein College of Medicine, Bronx, New York, United States of America; Claremont Colleges, UNITED STATES

## Abstract

Bayesian models have proven effective in characterizing perception, behavior, and neural encoding across diverse species and systems. The neural implementation of Bayesian inference in the barn owl’s sound localization system and behavior has been previously explained by a non-uniform population code model. This model specifies the neural population activity pattern required for a population vector readout to match the optimal Bayesian estimate. While prior analyses focused on trial-averaged comparisons of model predictions with behavior and single-neuron responses, it remains unknown whether this model can accurately approximate Bayesian inference on single trials under varying sensory reliability, a fundamental condition for natural perception and behavior. In this study, we utilized mathematical analysis and simulations to demonstrate that decoding a non-uniform population code via a population vector readout approximates the Bayesian estimate on single trials for varying sensory reliabilities. Our findings provide additional support for the non-uniform population code model as a viable explanation for the barn owl’s sound localization pathway and behavior.

## Introduction

Bayesian models have been successful in describing perception, behavior, and neural coding in multiple species and systems, and multiple theories exist for the neural implementation of this probabilistic inference [1–8]. Theories differ in multiple ways, including the type of probabilistic information that is represented in the brain and the neural mechanisms for encoding and decoding probabilistic information. A requirement for any model to explain the neural implementation of Bayesian perception or behavior is to represent sensory uncertainty on single trials.

**Fig 1:**
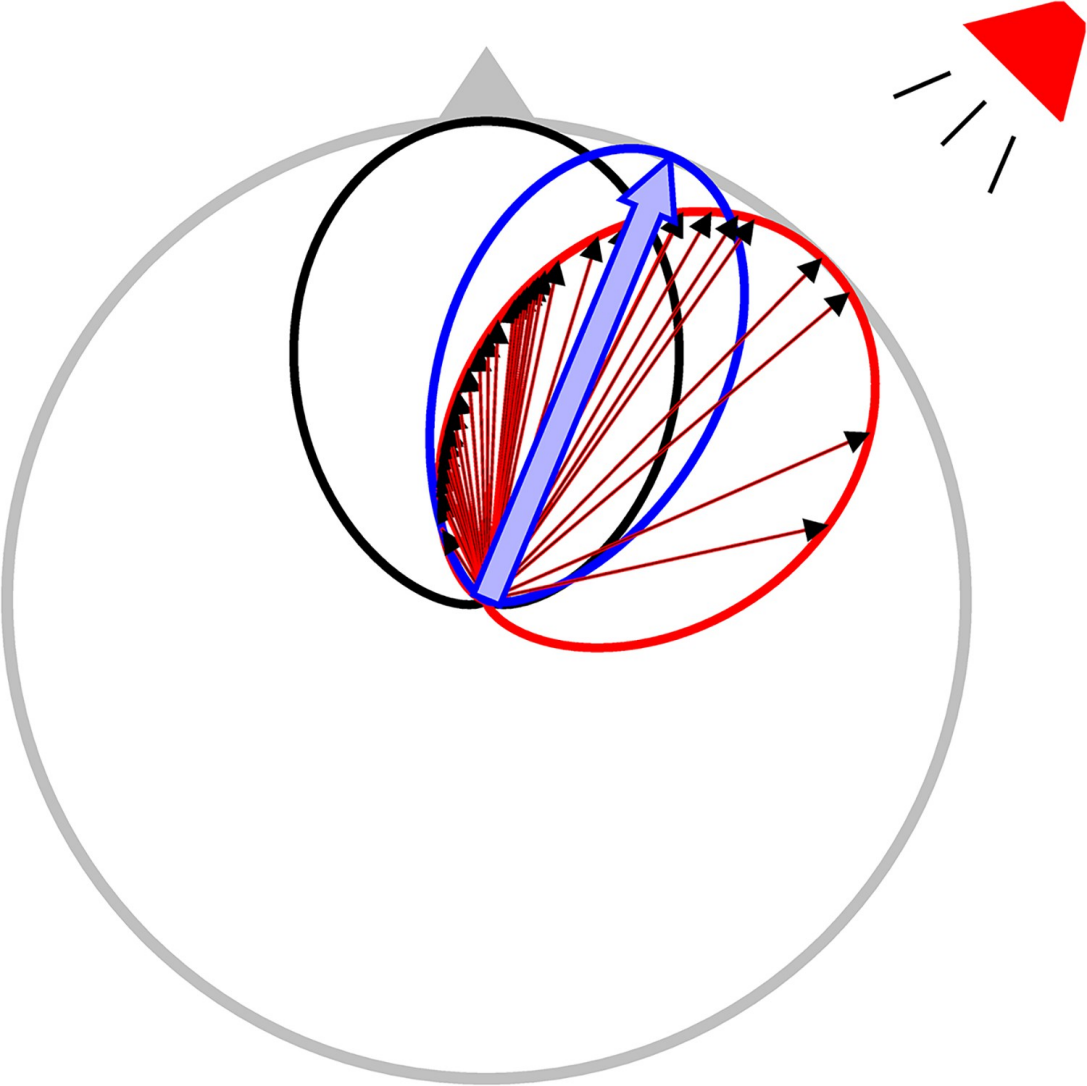
Non-uniform population code for Bayesian inference. The Bayesian estimate of sound direction is the mean of the posterior distribution (blue curve), which is determined by a product of the prior distribution of direction (black curve) and the stimulus-dependent likelihood (red curve). The non-uniform population code shows that a population vector readout (blue arrow) will match the Bayesian estimate when the preferred directions of neurons in the barn owl’s midbrain sound localization map are drawn from the prior distribution and their firing rates in response to a sound follow the pattern of the likelihood. Smaller arrows (red and black) represent the responses of a population of midbrain neurons that match the non-uniform population code, where the arrow directions indicate the neurons’ preferred directions and the lengths are proportional to the neurons response at the indicated stimulus direction (speaker symbol). These weighted cell vectors are combined to produce the population vector.

The barn owl’s sound localization system has been modeled as performing Bayesian inference using a non-uniform population code [5, 9, 10] (Fig 1). Experimental evidence for the model’s predictions has been demonstrated through neuronal and behavioral responses [11–15]. Previous studies showed that this model describes the barn owl’s trial-averaged sound localization behavior in azimuth [5], elevation [9], and as sensory reliability decreases [5, 14]. The model for the neural representation effectively predicted across-trial averaged neural responses where a population vector readout will match a Bayesian estimate [5, 12, 14] and the owl’s sound localizing behavior [5, 13]. In natural conditions, sensory noise in sound localization can change rapidly with the amount of background noise in the environment. A system performing optimal Bayesian inference of a target sound location should be able to weight the evidence from sound localization cues based on the current noise level.

While recent findings showed that trial-by-trial population responses of midbrain’s optic tectum (OT), a homologous region of the mammalian superior colliculus with topographically arranged spatial tuning [16, 17], match the pattern of activity predicted by the non-uniform population code model [14], it has not been tested whether a population vector readout of the non-uniform population can produce estimates that match the Bayesian estimate on single trials. The empirical study of Ferger et al. [14] supported a key prediction of the non-uniform population code model, but did not provide a theoretical justification that the non-uniform population code model will produce direction estimates that match the Bayesian estimate on single trials, nor did it explore the accuracy of this match for different population sizes. Based on this open question, previous experimental findings, and alternative models, we present new results demonstrating that the non-uniform population code produces estimates that match the Bayesian estimate on single-trial responses.

## Materials and methods

### Bayesian model

The methods follow the approach described in Fischer and Peña (2011). We model the prior distribution *p*(*θ*) of the sound source direction as a Gaussian distribution centered at 0 degrees with a standard deviation of 23.3 degrees. This distribution reflects the tendency for barn owls to spend more time facing prey during active prey capture [18]. The interaural time difference (ITD) in the auditory input, which is used to infer sound location, is modeled as a sinusoidal function of the sound source azimuth plus Gaussian noise ITD=Asin(ωθ)+η. The parameters *A* = 260 *μs* and *ω* = 0.0143 radians per degree are determined from the measured relationship between sound source direction and ITD measured from broadband frequency signals recorded at a barn owl’s ears [5, 19]. The amplitude *A* = 260 *μs* is the maximum ITD and the frequency *ω* = 0.0143 radians per degree determines direction at which the maximum ITD is produced. The noise *η* for fully binaurally correlated (binaural correlation = 1) broadband stimuli is assumed to have a Gaussian distribution with zero mean and standard deviation 41.2 *μs* [5]. Therefore, for a given direction, the conditional probability *p*(*ITD*|*θ*) is a Gaussian distribution with mean *A*sin(*ωθ*) and standard deviation 41.2 *μs*. The mean of the posterior distribution p(θ|ITD)∝p(ITD|θ)p(θ) is taken as the optimal Bayesian estimate in this analysis. The mean is computed from the direction of the Bayes’ vector $BV(ITD)=\int_{-\pi }^{\pi }p(\theta |ITD)u(\theta )d\theta$, where *u*(*θ*) is a unit vector pointing in the direction *θ*.

### Stimulus reliability

In behavioral experiments with barn owls, the reliability of ITD can be changed on single trials by varying the binaural correlation (BC) of the sounds at the two ears [20]. We previously used a cross-correlation model of the neural computation of ITD to estimate the relationship between BC and the standard deviation of ITD computed from the peak in the cross-correlation of the left and right auditory input signals [5]. Based on this result, we model the standard deviation of the noise corrupting ITD as an exponential function of BC

$$SD(BC)=41.2+219.34e^{-11.31\times BC}.$$

The Bayesian estimate is computed from the posterior distribution over direction given the ITD and BC computed from the left and right auditory input signals,

p(θ|ITD,BC)∝p(ITD|θ,BC)p(θ).

The assumption of conditioning on BC is reasonable, based on several simplifications that we have made. A more realistic formulation of the inference problem is to estimate the sound source direction from the time-dependent signals measured at the left and right ears, *s*_*L*_(*t*) and *s*_*R*_(*t*), respectively. In this case, the Bayesian estimate is the mean of the posterior distribution conditioned on these signals *p*(*θ*|*s*_*L*_(*t*),*s*_*R*_(*t*)). This distribution is equivalent to a distribution that also conditions on any function *F*(*s*_*L*_(*t*),*s*_*R*_(*t*)) of the left and right ear input signals ${p(\theta |s}_{L}(t),s_{R}(t),F(s_{L}(t),s_{R}(t)))$. One possible function is the cross-correlation of the left and right ear input signals, which provides an estimate of BC. Our simple model assumes that the ITD estimated from the cross-correlation carries the relevant information about the source azimuth that is present in the input signals. In experimental conditions that use several BC values with a coarse resolution, the BC can be estimated from the cross-correlation of the left and right ear input signals with enough accuracy that it can be assumed known [20]. We therefore model the ITD likelihood *p*(*ITD*|*θ*,*BC*) as a Gaussian function of ITD with a standard deviation that depends on the stimulus BC, which is consistent with experimental observations [14].

### OT population model

The model optic tectum population consists of *N* direction selective neurons that respond to the stimulus ITD and BC. The preferred directions *θ*_*n*_ are drawn from the Gaussian prior distribution *p*(*θ*) (see above). The response of a neuron with preferred direction *θ*_*n*_, denoted *r*_*n*_(*ITD*(*θ*)), is drawn from a Poisson distribution with a mean that is proportional to the ITD likelihood function, $a_{n}(ITD(\theta ))=r_{max}p(ITD|\theta_{n})$. While the Poisson noise is independent across neurons, the neurons exhibit correlated variability due to the input noise corrupting ITD that is shared across neurons. Maximum firing rates of OT neurons decrease as BC decreases [12, 14, 20, 21]. We thus modeled the maximum firing rate as a quadratic function of BC, $r_{max}=4.9{BC}^{2}+1.2BC+0.3,$ to match experimental observations [20]. Note that the decrease in firing rate at low BC scales the population activity uniformly and does not represent uncertainty in the non-uniform population code model.

The population vector decoder is a linear combination of the preferred direction vectors:

$$PV(ITD(\theta ))=\frac{1}{N}\sum_{n=1}^{N}r_{n}(ITD(\theta ))u(\theta_{n})$$

where *u*(*θ*_*n*_) is a unit vector pointing in the direction *θ*_*n*_. The direction estimate from the OT population response is the direction of the population vector. For comparison of the population vector model with the Bayesian model, the same randomly generated ITD is used as input to both models on each trial.

## Results

Given the different ways to refer to Bayesian models in the literature, we start by reviewing the framework in which we are comparing a neural decoder to a Bayesian estimate. Here, we focus on the barn owls’ localization of azimuthal direction of a sound source θ based on the interaural time difference (ITD) from sound signals arriving at left and right ears [22]. An ideal observer uses the measured ITD to estimate the direction *θ*. In the Bayesian framework, the environmental variable *θ* and the sensory information ITD are characterized by probability distributions that determine the form of the optimal estimate of the direction *θ*. A Bayesian estimate is produced from the posterior distribution p(*θ*|*ITD*), which describes the distribution of the direction variable after the sensory input ITD has been measured. Bayes’ theorem states that the posterior distribution can be defined as p(θ|ITD)∝p(ITD|θ)p(θ), where the prior distribution *p*(*θ*) is the probability distribution of θ before sensory information has been received and the likelihood *p*(*ITD*|*θ*) represents how the sensory input ITD depends on the sound source direction θ. A full probabilistic model is thus determined by specific prior and likelihood models. The Bayesian estimate used here for each single trial is the mean of the posterior distribution *p*(*θ*|*ITD*). The challenge for a neural code to approximate Bayesian inference in this context, therefore, is to specify a neural representation and decoder that closely approximates mean of the posterior distribution *p*(*θ*|*ITD*) for each stimulus presentation.

The non-uniform population code model describes conditions on the pattern of activity over a neural population that allows a population vector decoder to match the optimal Bayesian estimate, as predicted for barn owl sound localization [5] and human visual orientation [23]. We assume that the direction estimate is decoded from a population of *N* neurons that respond to the stimulus ITD on a single trial with spike counts $r_{n}(ITD(\theta )),n=1,2,\ldots ,N$ using a population vector decoder,

$$PV(ITD(\theta ))=\frac{1}{N}\sum_{n=1}^{N}r_{n}(ITD(\theta ))u(\theta_{n})$$

where *u*(*θ*_*n*_) is a unit vector pointing in the direction *θ*_*n*_. The direction *θ*_*n*_ is called the preferred direction of the *n*^*th*^ neuron. The spike counts *r*_*n*_(*ITD*|*θ*)) are drawn from a neural noise distribution, here assumed to be Poisson, with rates *a*_*n*_(*ITD*|*θ*)). We have shown that if the preferred directions *θ*_*n*_ are drawn independently from the prior distribution *p*(*θ*) and the pattern of rates over the population is proportional to the likelihood $a_{n}(ITD(\theta ))\propto p(ITD|\theta_{n})$, then the trial-averaged population vector estimate will converge to the Bayesian estimate as the number of neurons gets large [5, 9]. Here we show that, additionally, the single-trial population vector estimates can accurately approximate the Bayesian estimate.

### Single-trial Bayesian decoding: Simulation

**Fig 2:**
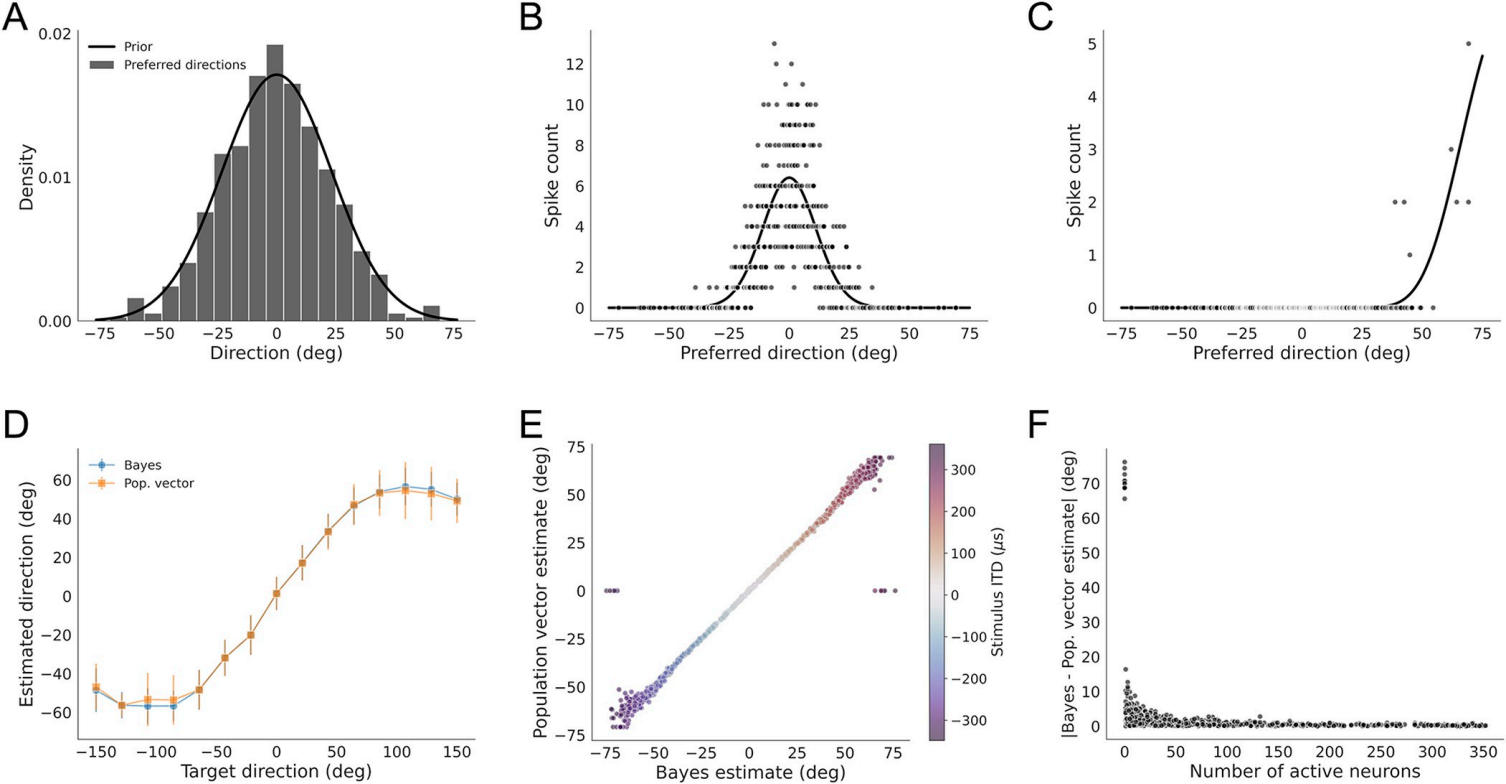
Single trial decoding in a small population (n = 500). (A) Distribution of preferred directions in a population of 500 model neurons, as used in Fischer and Peña, 2011. (B, C) Example single-trial population responses to stimuli with ITD = 0 μs (B) and ITD = 250 μs (C). The solid line is proportional to the likelihood function, which determines the firing rates of the neurons. BC = 1 for all stimuli. (D) Trial averaged comparison of the Bayesian direction estimate (blue) and the population vector decoder estimate (orange) as a function of target direction, as in Fischer and Peña 2011 Fig 1E. (E) Single-trial comparison of the Bayesian direction estimate and the population vector decoder estimate across stimulus direction. The color codes the stimulus ITD. (F) Difference between the Bayesian direction estimate and the population vector decoder estimate vs. the number of active neurons. Larger errors occur for small number of active neurons and the largest errors occur when no neurons respond to a peripheral stimulus.

We first show that population vector estimates from the original non-uniform population code model for the barn owl’s optic tectum (OT) [5] approximate the Bayesian estimate of sound source direction on single trials. This OT model has 500 neurons with preferred directions drawn from a Gaussian distribution matching the prior distribution over direction (Fig 2A), which are the same parameters used in the model of Fischer and Peña (2011). We tested this model for a range of stimulus directions, using 50 trials at each direction. For a given stimulus direction, the ITD varies across trials due to noise corrupting the sinusoidal mapping from direction to ITD. This noise varies with the binaural correlation between the left and right sound signals, but in this example we consider fully correlated sounds (BC = 1). The neural responses on each trial are drawn from a Poisson distribution where the rates are proportional to the likelihood function determined by the ITD on that trial (Fig 2B and 2C). Consistent with previous results (Fischer and Peña, 2011 Fig 1E), the trial-averaged population vector estimates closely approximated the Bayesian estimates (Fig 2D). In addition, the single-trial population vector estimates closely approximated the Bayesian estimates for most stimuli, with some large errors (Fig 2E) observed only for stimuli near the side of the head, which result in responses of a few or zero neurons to the stimulus in this model (Fig 2C and 2F). The largest deviations occurred due to small numbers of neurons responding to the stimulus, a consequence of the small model population size of 500, which is far lower than the number of neurons in the OT [24]. Nevertheless, this analysis shows that the conditions specified in the non-uniform population code produce a neural model where population vector estimates can closely approximate the Bayesian estimate on single trials.

### Accuracy and population size

**Fig 3:**
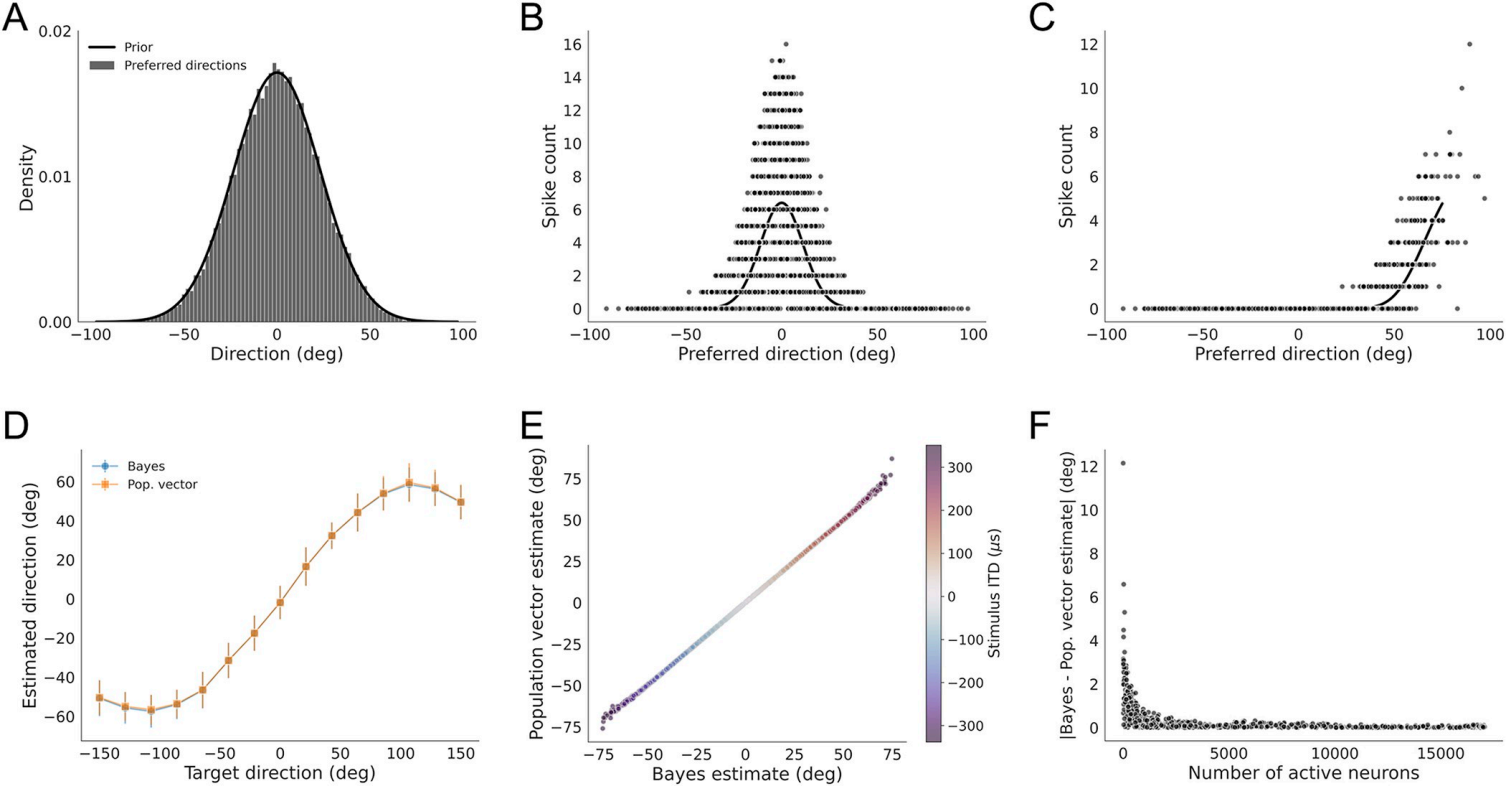
Single trial decoding in a large population (n = 25,000). Same scheme as Fig 2, indicating the distribution of preferred directions in a population for a population of 25,000 neurons (A), single-trial population responses to stimuli with ITD = 0 μs (B) and ITD = 250 μs (C), trial averaged comparison of the Bayesian direction estimate (blue) and the population vector decoder estimate (orange) as a function of target direction (D), single-trial comparison of the Bayesian direction estimate and the population vector decoder estimate across stimulus direction (E, color code of stimulus ITD indicated), and the difference between the Bayesian direction estimate and the population vector decoder estimate vs. the number of active neurons (F). Note errors in the periphery are smaller than in Fig 2 and large errors do not occur because there are always at least a few neurons responding to the stimulus.

We next showed that the population vector accurately approximates the Bayesian estimate on single trials for source directions around the head for a larger population. It is known that the accuracy of the trial-averaged population vector approximation to the Bayesian estimate increases with population size [5]. To test the approximation on single trials, we repeated the decoding analysis shown in Fig 2 for a model population of 25,000 neurons (Fig 3). The preferred directions were drawn from the same prior distribution as in the 500-neuron model (Fig 3A). As before, the neural responses on each trial were drawn from a Poisson distribution where the rates are proportional to the likelihood function determined by ITD computed from the stimulus on that trial (Fig 3B and 3C). In this larger model population, there are more neurons responding to stimuli in the periphery and thus the pattern of activity more accurately reflects the shape of the likelihood function (Fig 3C) than it does for the smaller modeled population (Fig 2C). Correspondingly, the population vector closely approximates the Bayesian estimate on average (Fig 3D) and on single trials (Fig 3E) for all stimulus directions. For this larger example population, the differences between the population vector and Bayesian estimates were less than 12.15 degrees (Fig 3F). Similar to what was observed for the smaller population, the error larger than 10 degrees occurred in this larger model population when only two neurons responded to a peripheral stimulus where few neurons have nearby preferred directions (Fig 3F). This example illustrates that the non-uniform population code can produce decoded direction estimates that closely approximate the Bayesian estimate for single trials.

**Fig 4:**
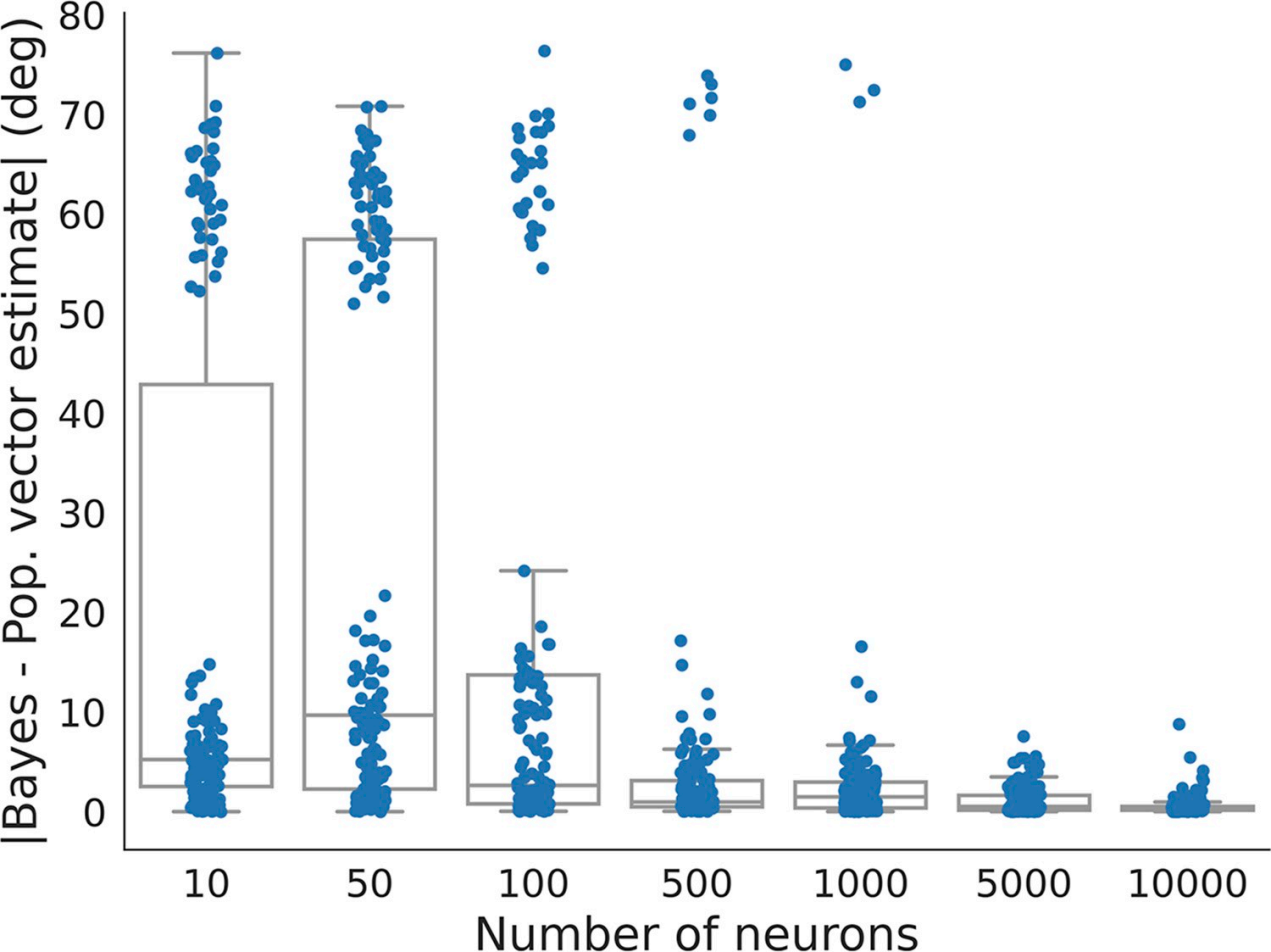
Single trial error vs. population size. Errors between the Bayesian direction estimate and the population vector decoder estimate over directions between -90 and 90 degrees vs. the number of neurons in the population. At each population size, the boxplots show the first quartile, median, and third quartile with the box, and whiskers extending to the minimum and maximum errors that are not outliers. The large errors that occur because of a lack of responding neurons were not observed for populations of approximately 5,000 or more neurons.

We evaluated the performance of the neural decoding at different population sizes, taking into account the effects of random selection of preferred directions from the prior, noise in the mapping from direction to ITD, and Poisson neural noise. To quantify the accuracy of the population vector approximation to the Bayesian estimate, we tested different realizations of these random elements at each population size. The maximum single-trial difference between the population vector and Bayesian estimates was greater than 70 degrees for population sizes less than 5,000 neurons (Fig 4). As shown in the example in Fig 2, the large differences occurred when there were zero neurons responding to the stimulus. The median single-trial difference between the population vector and Bayesian estimates decreased as the number of neurons increased (Fig 4). This shows that the accuracy of the population vector approximation to the Bayesian estimate improves with increasing population size, where the approximation error is within in several degrees for directions covering the frontal hemisphere for population sizes greater than approximately 5,000 neurons. This is smaller than the number of neurons in the midbrain auditory space map. The midbrain auditory space map arises in the external nucleus of the inferior colliculus (ICx), where the volume of the nucleus and the density of cells suggests that ICx contains more than 50,000 neurons [24]. The auditory space map in OT results from point-to-point projections from ICx [25] and the volume of OT is approximately four times larger than the volume of ICx [26], suggesting that OT contains many more ITD-sensitive neurons than needed to achieve accurate approximation of the Bayesian estimate by the population vector. The provided supporting information contains a proof (S1 Appendix) that the population vector will converge to the Bayesian estimate as the number of neurons grows.

### Coding stimulus reliability

**Fig 5:**
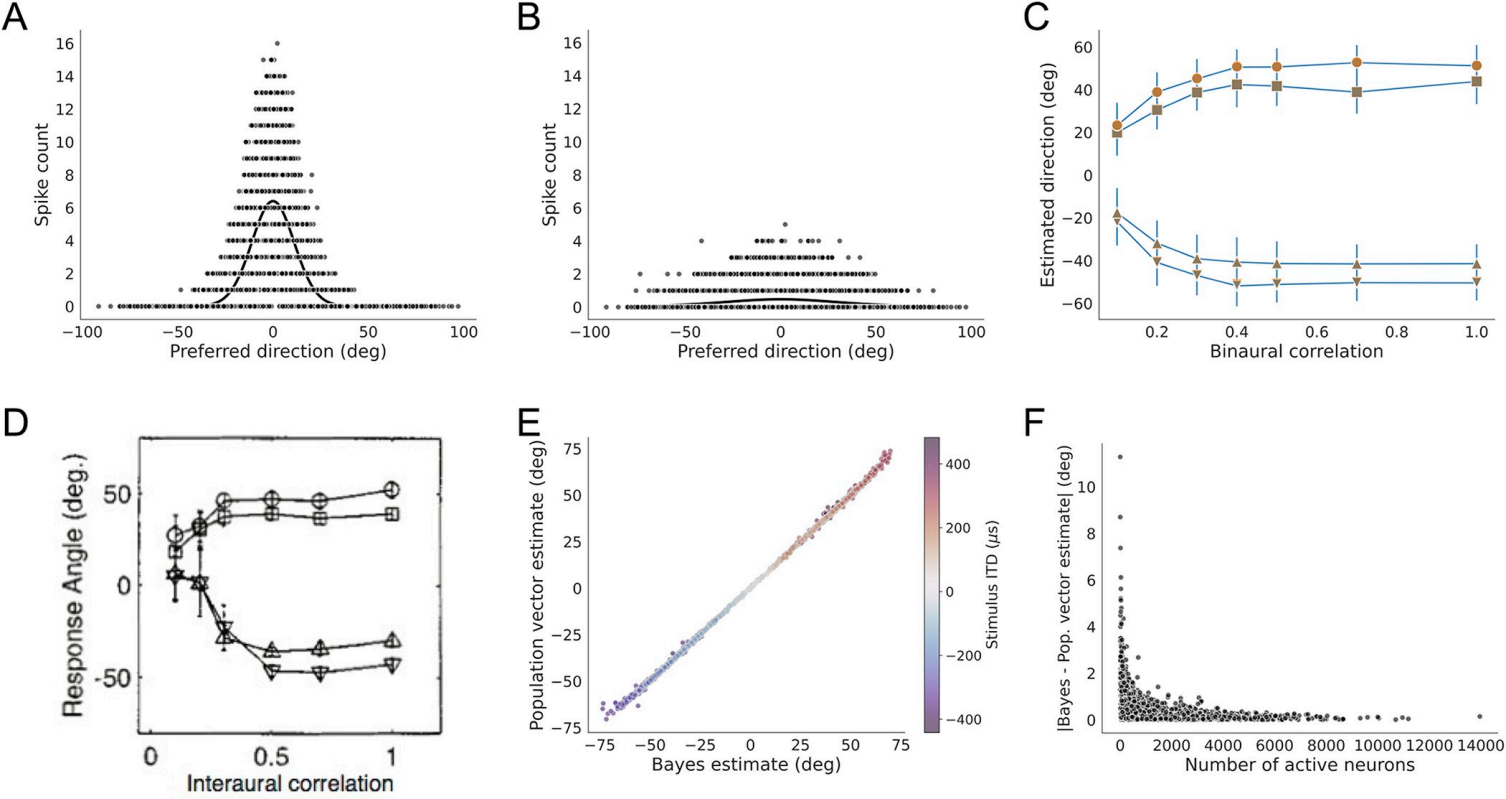
Single trial decoding across manipulated stimulus reliability. (A, B) Example single-trial population responses to stimuli with BC = 1 (A) and BC = 0.1 (B). The solid line is proportional to the likelihood function. (C) Trial averaged comparison of the Bayesian direction estimate (blue) and the population vector decoder estimate (orange) for example stimulus directions of 75 (circles), 55 (squares), -55 (triangles), -75 (inverted triangles) degrees as a function of BC, as in Fischer and Peña (2011) Fig 2B. (D) A barn owl’s behavior under the same stimulus conditions reproduced from [20]. (E) Single-trial comparison of the Bayesian direction estimate and the population vector decoder estimate combined over stimulus direction and BC. The color represents the stimulus ITD on each trial. (F) Difference between the Bayesian direction estimate and the population vector decoder estimate vs. the number of active neurons combined over stimulus direction and BC. The largest errors occur when no neurons respond to the stimulus in which case the estimate is assumed to be zero.

The non-uniform population code can accurately approximate the Bayesian estimate for different levels of stimulus reliability on single trials. In behavioral experiments with barn owls, the reliability of ITD is changed on single trials by varying the binaural correlation (BC) of the sounds at the two ears [20]. We simulated the manipulation of the reliability of ITD by varying BC using a non-mechanistic model where the standard deviation of the noise corrupting ITD depends on BC. We previously used a cross-correlation model of the neural computation of ITD to estimate the relationship between BC and the standard deviation of the ITD computed from the left and right auditory input signals [5]. Following Fischer and Peña (2011), we modeled the standard deviation of the noise corrupting ITD as an exponential function of BC. Corresponding to the increased standard deviation of the sensory noise as BC decreases, the likelihood function in the Bayesian model gets wider as BC decreases (Fig 5A–5C). As a result, the Bayesian estimate is increasingly biased toward the front as BC decreases, which matches the barn owl’s behavior [5, 20] (Fig 5D). We used the 25,000-neuron model to test the accuracy of single-trial population vector decoding matching the Bayesian estimate as sensory reliability varies. Again, the neural responses on each trial were drawn from a Poisson distribution where the rates are proportional to the likelihood function determined by the ITD on that trial. Thus, the pattern of activity broadens over the population as BC decreases, consistent with measurements made in the OT with multielectrode arrays [14]. In addition, we model the maximum firing rate over the population as a quadratic function of BC to model the decrease in firing rate as BC decreases [12, 14, 20, 21]. With this model, the population vector accurately approximated the Bayesian estimate on average (Fig 5C) and on single trials (Fig 5E). Both the barn owl’s behavior and the population vector model show an underestimation of the source direction at all BC values, as predicted by the Bayesian model [5]. As reported above, the largest errors occurred when few or zero neurons responded to the stimulus (Fig 5F). While both the width of population activity and the maximum firing rate change with BC, it is the width of population activity that determines the underestimation of the population vector estimate (S1 Appendix). The accurate approximation of the Bayesian estimate by the population vector as BC changes is expected because the model satisfies the assumption that the neural activity pattern matches the likelihood, which widens as BC decreases.

## Discussion

The barn owl’s sound localization system has successfully been modeled as performing Bayesian inference using a non-uniform population code [5, 9, 10, 12, 14, 15]. Here we used analysis and simulations to show that decoding a non-uniform population code with a population vector accurately approximates a Bayesian estimate on single trials for different levels of sensory reliability. This work further supports this model for the barn owl’s sound localization pathway and behavior.

Recent multielectrode array recordings support the prediction of the model that the spread of population activity on single-trials represents sensory reliability [14]. Simultaneous recordings of multiple units across the OT space map show remarkable agreement with the pattern of activity predicted by the model for correlated and decorrelated sounds on single trials. Previous comparisons of the model to neurophysiological data relied on trial-averaged tuning curves [5, 9, 12] to infer the spread of activity over the population because array recordings were not available. While previous work focused on the trial-averaged results, the models used in previous work function consistently with the model used here to demonstrate the neural implementation of Bayesian inference on single trials. The multielectrode array recordings [14] and modeling presented here support the non-uniform population code model of optimal inference of sound location in the barn owl’s midbrain sound localization pathway.

Here we explored the neural implementation of Bayesian inference on single trials where sensory noise varies by changing BC. Changing sensory noise alters the width of the likelihood function of the Bayesian model, but other changes to the likelihood or prior are possible. For example, the mean of the likelihood can change by altering the mapping from directions in space to ITD. This occurs naturally during growth and has been experimentally manipulated via ear occlusion, or external ear modification [15, 27–30]. The prior distribution may change when the most common locations of relevant sound sources in the environment changes. This could occur through changes in the physical configuration of the environment or through a change in the owl’s hunting strategy that places the owl in different positions relative to prey. The prior distribution for directions in visual space has been experimentally manipulated via prism glasses [27, 31]. It is therefore important to address the flexibility of both the non-uniform population code model and the owl’s sound localization system to respond to these changes to determine under what conditions Bayesian integration reflecting changing environmental statistics may be maintained.

The extensive literature on learning in the owl’s sound localization system shows that the owl’s sound localization behavior and neural responses in the sound localization pathway respond to changes in the environment over multiple time scales. For example, when the sensory noise level changes by varying BC, neural responses and the owl’s behavior change on a trial-by-trial basis [14, 20]. The widening of population activity in the auditory space map that is predicted by the non-uniform population model results from the cross-correlation operation used to compute ITD in the sound localization pathway and therefore does not require changes to circuit connectivity to be implemented [32]. Thus, the cross-correlation operation produces the change in response with BC such that the activity pattern in the auditory space map matches the likelihood. However, when the mapping between sound localization cues and directions in visual space is disrupted by prism glasses, ear occlusion or modification, owls that experience these changes early in life learn to compensate and ultimately learn new neural selectivity for sound localization cues in the auditory space map after several weeks [15, 27]. These experimental manipulations alter the mean ITD and the pattern of ITD reliability across frequency at each direction, and the resulting changes in ITD and frequency selectivity of midbrain space map neurons demonstrates a long-term change of the likelihood in the barn owl’s midbrain [15, 27]. These studies show that a change in the likelihood function does not lead to an instantaneous change in the neural representation of sounds. Nevertheless, it is possible to reorganize the inputs to the auditory space map, at least within critical periods of development, to adapt to changes in the likelihood.

In the non-uniform population code model, the prior is implemented through the distribution of preferred directions of the population of neurons. The use of prisms to displace the visual field causes a stable change to the prior for directions in visual space. As discussed above, when experienced in early life, owls learn new neural selectivity for sound localization cues in the auditory space map after several weeks in response to this change in the prior [27]. A long-term change in the prior in the non-uniform population code could be implemented through changes in the synaptic weights between the midbrain auditory space map neurons and their inputs [27] and downstream targets [13]. Changing the prior in the Bayesian model that the PV estimate in the non-uniform population code matches over a rapid time scale does not require a change in the preferred directions, but can be implemented rapidly using gain modulation of population responses [33]. If a stimulus-independent gain that is equal to a ratio of the new prior to the old prior multiplies the population responses, then the PV will approximate a Bayesian estimate under the new prior (S1 Appendix). There is experimental evidence that top-down modulatory signals can control the gain of neurons in the midbrain auditory space map on a rapid time scale [34, 35], but these experiments did not manipulate a prior for direction. The non-uniform population code model has the theoretical capability to flexibly implement Bayesian inference on single trials in an environment with changing statistics, but further work is required to explore the limits of this flexibility in the barn owl’s sound localization system.

There are alternative models for the neural implementation of Bayesian inference that have been proposed in the literature. The sampling hypothesis proposes that stimulus-driven neural activity represents samples from a posterior distribution [4, 36, 37]. This type of code may allow for flexible neural implementation of Bayesian inference, but it has been shown to be inconsistent with responses of neurons in the barn owl’s auditory space map [38]. It has been proposed that top-down modulation of bottom-up sensory input may support Bayesian integration of sensory and prior information in visual cortex [39]. This is consistent with our proposal that top-down modulation of midbrain responses could modify the prior in the non-uniform population code [33] (S1 Appendix). However, the robust localization performance of barn owl after inactivation of the forebrain pathway, which is likely the source of top-down modulatory inputs, suggests that top-down modulation does not consistently supply a prior in the owl’s sound localization system [40]. Alternatively, behavior that is consistent with Bayesian inference could be produced through a mechanism that does not perform Bayesian integration, but rather implements an approximation that is sensitive to the level of sensory noise. This could occur by modifying the synaptic weights between the midbrain auditory space map neurons and their downstream targets as sensory noise changes [13, 41], or through population dynamics that change with sensory noise to yield location estimates that reflect Bayesian inference [8, 42]. Further experiments are required to test these hypotheses in the barn owl’s sound localization system.

The work here highlights contrasts between the non-uniform population code model and a linear probabilistic population code (PPC) model of inference in the barn owl’s sound localization system [43]. The non-uniform population code model makes use of the overrepresentation of frontal space that is observed in the OT [16] to implement the owl’s behavioral bias for sounds located in peripheral locations, which is already observed for perfectly correlated [13, 19, 44] and increased for both decorrelated and high frequency sounds [20]. The linear PPC model of [43], in contrast, reported little difference between flat or Gaussian prior distributions and did not match the normal owl’s localization frontal bias observed for highly correlated inputs from sounds in peripheral locations. Additionally, a non-flat prior in a linear PPC would require the presence of a population providing input to OT that carries information about the prior [3] and there is no experimental evidence for such a source of a prior. Given that the nature of the prior (the distribution of prey locations during prey capture) is evolutionarily based, a prior encoded in the preferred direction of neurons is a more efficient representation of stimuli than a permanently active neural input [45]. Recent reports suggesting that auditory responses in the barn owl’s midbrain are shaped by the reliability of ITD experienced early in development, rather than ongoing evidence, support this claim [11, 15].

In summary, the modeling work presented here, in conjunction with electrophysiological work [14], supports the efficacy of a non-uniform population code to compute sound location in the barn owl’s midbrain. This extends the theory that Bayesian priors can be represented in the preferred stimuli of neurons and the statistics of sensory cues can be represented in the response patterns of populations of neurons. This non-uniform population code is general and may be implemented in different sensory systems, when priors can be expected to be stable over time.

## Supporting information

S1 Appendix Single-trial Bayesian decoding: Theory. (PDF)
