# Supplementary material for: Single trial Bayesian inference by population vector readout in the barn owl’s sound localization system
Source: PLoS One. 2024 May 21;19(5):e0303843. doi: 10.1371/journal.pone.0303843 (PMC11108143; doi:10.1371/journal.pone.0303843)
Supplement: S1 Appendix — (PDF) [file pone.0303843.s001.pdf]

# Supporting Information

## S1 Appendix

## Single-trial Bayesian decoding: Theory

We present a proof that if the preferred directions $\theta_n$ are drawn independently from the prior distribution $p(\theta)$ and the pattern of rates over the population is proportional to the likelihood $a_n(ITD(\theta)) \propto p(ITD|\theta_n)$, then the single-trial population vector estimate will converge to the Bayesian estimate as the number of neurons grows to infinity.

The population vector is an average of weighted preferred direction vectors

$$PV(ITD(\theta)) = \frac{1}{N}\sum_{n=1}^{N} r_n\big(ITD(\theta)\big)\, u(\theta_n)$$

where the weights are the neural responses $r_n\big(ITD(\theta)\big)$. For a given ITD, the neural responses are assumed to be independent Poisson random variables

$$r_n(ITD(\theta)) \sim \mathrm{Poisson}(a_n(ITD(\theta)))$$

with mean values $a_n(ITD(\theta))$. The non-uniform population code proposes that the pattern of mean values across the population is proportional to the likelihood function

$$a_n(ITD(\theta)) = r_{max}\, p(ITD(\theta)|\theta_n)$$

and the preferred directions are drawn independently from the prior over direction $\theta_n \sim p(\theta)$. Under these assumptions, for a given ITD, the population vector is an average of independent and identically distributed random vectors $X_n$:

$$PV(ITD(\theta)) = \frac{1}{N}\sum_{n=1}^{N} r_n(ITD(\theta))u(\theta_n) = \frac{1}{N}\sum_{n=1}^{N} X_n$$

where $X_n = r_n(ITD(\theta))u(\theta_n)$. The distribution of the random vectors $X_n$ depends on the prior over direction, from which the preferred directions are drawn, and the Poisson neural noise distribution. The conditional expected value $E[X_i|ITD]$ is proportional to the Bayes' direction vector:

$$\begin{aligned}
E[X_i|ITD] &= E_\Theta E_{X|\Theta}[r_i(ITD)u(\theta_i)|ITD] \\
&= E_\Theta[a_i(ITD)u(\theta_i)|ITD] \\
&= E_\Theta[r_{max}p(ITD|\theta_i)u(\theta_i)|ITD] \\
&= r_{max}\int_{-\pi}^{\pi} p(\theta)p(ITD|\theta)u(\theta)d\theta \\
&= \mu \\
&\propto r_{max}\int_{-\pi}^{\pi} \frac{p(\theta)p(ITD|\theta)}{p(ITD)}u(\theta)d\theta \\
&\propto BV(ITD)
\end{aligned}$$

where the Bayes' direction vector is given by

$$BV(ITD) = \int_{-\pi}^{\pi} p(\theta|ITD)u(\theta)d\theta.$$

Therefore, the law of large numbers shows that the population vector will converge to a vector pointing in the same direction as the Bayes' vector as the population size grows to infinity

$$PV(ITD(\theta)) = \frac{1}{N}\sum_{n=1}^{N} r_{in}(ITD(\theta))u(\theta_n) = \frac{1}{N}\sum_{n=1}^{N} X_n \to \mu \propto BV(ITD).$$

This shows that the single-trial population vector will converge to the Bayesian estimate in the limit of an infinite population size.

This argument uses the assumption that the noise distributions in the neural activities are conditionally independent given the ITD. The argument does not, however, depend on the assumption of a Poisson noise distribution and would remain valid for alternative noise distributions. This shows that the result is highly robust to the level of neural noise in the responses. This work also lets us determine how robust the population vector estimates will be to deviations of the parameters, such as the likelihood or prior mean and variance, from the desired values. The population vector will approximate a Bayesian estimate from a model with a prior matching the distribution of preferred directions and a likelihood matching the shape of the population response. Finally, although we use example of estimating azimuth from ITD, the result generalizes to estimating azimuth and elevation from a spectrum of ITD and interaural level difference cues [1].

## Gain control to modify the prior: Theory

We now show how a gain modulation of the population responses can alter the prior [2]. As before, assume that the preferred directions $\theta_n$ are drawn independently from a prior

distribution $p(\theta)$. If the pattern of rates over the population is proportional to the likelihood multiplied by the ratio of a new prior distribution $q(\theta)$ to the original prior distribution $p(\theta)$, $a_n(ITD(\theta)) \propto \frac{q(\theta_n)}{p(\theta_n)} p(ITD|\theta_n)$, then the single-trial population vector estimate will converge to the Bayesian estimate under the new prior distribution $q(\theta)$ as the number of neurons grows to infinity.

The population vector is an average of weighted preferred direction vectors

$$PV(ITD(\theta)) = \frac{1}{N} \sum_{n=1}^{N} r_n\big(ITD(\theta)\big)\, u(\theta_n)$$

where the weights are the neural responses $r_n\big(ITD(\theta)\big)$. For a given ITD, the neural responses are assumed to be independent Poisson random variables

$$r_n(ITD(\theta)) \sim \mathrm{Poisson}(a_n(ITD(\theta)))$$

with mean values $a_n(ITD(\theta))$. In this case, the pattern of mean values across the population is proportional to the likelihood function multiplied by the ratio of a new prior distribution $q(\theta)$ to the original prior distribution $p(\theta)$

$$a_n(ITD(\theta)) = r_{max} \frac{q(\theta_n)}{p(\theta_n)} p(ITD(\theta)|\theta_n)$$

and the preferred directions are drawn independently from the prior over direction $\theta_n \sim p(\theta)$. Under these assumptions, for a given ITD, the population vector is an average of independent and identically distributed random vectors $X_n$:

$$PV(ITD(\theta)) = \frac{1}{N} \sum_{n=1}^{N} r_n(ITD(\theta)) u(\theta_n) = \frac{1}{N} \sum_{n=1}^{N} X_n$$

where $X_n = r_n(ITD(\theta))u(\theta_n)$. The distribution of the random vectors $X_n$ depends on the prior over direction, from which the preferred directions are drawn, and the Poisson neural noise distribution. The conditional expected value $E[X_i|ITD]$ is proportional to the Bayes' direction vector:

$$E[X_i|ITD] = E_\Theta E_{X|\Theta}[r_i(ITD)u(\theta_i)|ITD]$$

$$= E_\Theta[a_i(ITD)u(\theta_i)|ITD]$$

$$= E_\Theta\left[r_{max}\frac{q(\theta_i)}{p(\theta_i)}p(ITD|\theta_i)u(\theta_i)\middle|ITD\right]$$

$$= r_{max}\int_{-\pi}^{\pi} p(\theta)\frac{q(\theta)}{p(\theta)}p(ITD|\theta)u(\theta)d\theta$$

$$= r_{max}\int_{-\pi}^{\pi} q(\theta)p(ITD|\theta)u(\theta)d\theta$$

$$= \mu$$

$$\propto r_{max}\int_{-\pi}^{\pi}\frac{q(\theta)p(\theta|ITD)}{p(ITD)}u(\theta)d\theta$$

$$\propto BV(ITD)$$

where the Bayes' direction vector is given by

$$BV(ITD) = \int_{-\pi}^{\pi}\frac{q(\theta)p(ITD|\theta)}{p(ITD)}u(\theta)d\theta.$$

Therefore, the law of large numbers shows that the population vector will converge to a vector pointing in the same direction as the Bayes' vector under the new prior distribution $q(\theta)$ as the population size grows to infinity

$$PV(ITD(\theta)) = \frac{1}{N}\sum_{n=1}^{N} r_{in}(ITD(\theta))u(\theta_n) = \frac{1}{N}\sum_{n=1}^{N} X_n \to \mu \propto BV(ITD).$$

This shows that the single-trial population vector will converge to the Bayesian estimate in the limit of an infinite population size.

## Gain control to modify the prior: Simulation

We used the network from Fig 3 to illustrate how gain modulation of the population response can alter the population vector to match a Bayesian estimate from a model with a prior that differs from the prior used to determine the distribution of preferred directions. Similar results have been shown for a center-of-mass decoder in [2]. The model population of 25,000 neurons has preferred directions that were drawn from the Gaussian prior distribution with mean 0 deg and standard deviation 23.3 deg, as used in Fig 3. We considered cases where prior in the Bayesian model is shifted laterally or made wider or narrower.

We first considered examples where the prior is shifted laterally. In the first example, the prior is shifted to be a Gaussian with mean -30 deg and standard deviation 23.3 deg (Fig S.1A). The gain multiplying the population spiking probability is the ratio of the new prior distribution to the original prior distribution, so the gain in this example is high for neurons with negative preferred directions (Fig S.1B). This produces a population response pattern that is shifted relative to the likelihood function in the direction of the new prior (Fig S.1C). As predicted by the theory, the population vector matches the Bayesian estimate from the model with the shifted prior (Fig S.1D and E). There are some errors for extreme positive directions where neurons do not respond to

the stimulus because of the gain. We next consider an example where the prior is shifted to be a Gaussian with mean 30 deg and standard deviation 23.3 deg (Fig S.1F). In this case, the gain is high for neurons with positive preferred directions (Fig S.1G) and the population response pattern is shifted relative to the likelihood function in the direction of the new prior (Fig S.1H). The population vector again matches the Bayesian estimate from the model with the shifted prior (Fig S.1I and J). Similar to the previous example, there are some errors for extreme negative directions where neurons do not respond to the stimulus because of the gain.

We also considered examples where the width of the prior changes, as considered for a center-of-mass decoder in [2]. In the first example of changing the width, the prior is wider than the original prior, being a Gaussian with mean 0 deg and standard deviation 35 deg (Fig S.1K). In this case, the gain is high for neurons with peripheral preferred directions (Fig S.1L) and the population response pattern is wider than the likelihood function (Fig S.1M). The population vector again matches the Bayesian estimate from the model with the shifted prior (Fig S.1N and O). In the second example of changing the width, the prior is narrower than the original prior, being a Gaussian with mean 0 deg and standard deviation 15 deg (Fig S.1P). In this case, the gain is high for neurons with central preferred directions (Fig S.1Q) and the population response pattern is narrower than the likelihood function (Fig S.1R). The population vector again matches the Bayesian estimate from the model with the shifted prior (Fig S.1S and T), with some errors for the most peripheral directions where neurons do not respond to the stimulus due to the gain that emphasizes central directions.

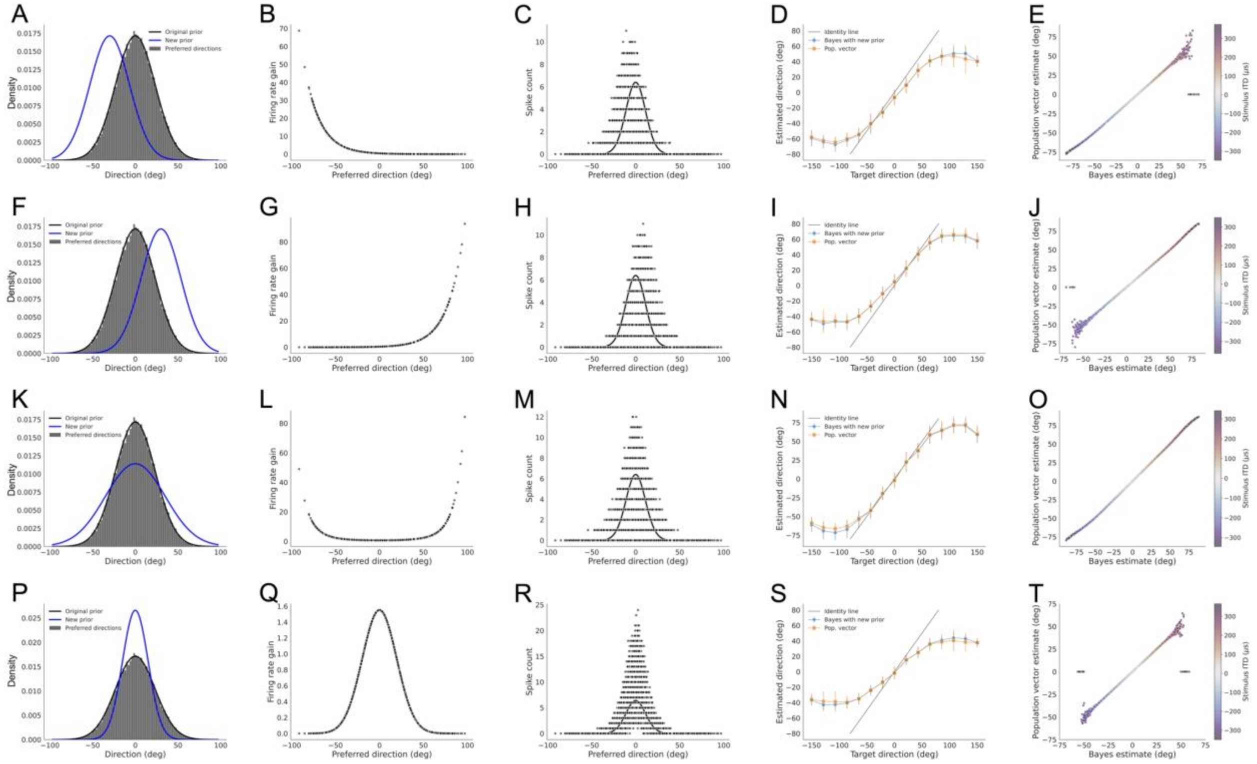


**Fig S.1. Changing the prior using gain modulation.** (A) The distribution of preferred directions in a population for a population of 25,000 neurons, along with the prior from which the preferred directions were drawn (black) and the new prior for the Bayesian model that the population vector (PV) estimates should match (blue). (B) Gain factor multiplying the population spiking probability that changes the PV to match Bayesian estimates from the model with the new prior. (C) Example single-trial population responses to stimuli with ITD = 0 µs and the likelihood function (solid line). (D) Trial averaged comparison of the Bayesian direction estimate (blue) and the population vector decoder estimate (orange) as a function of target direction. The black line is the identity line. Note that the direction with minimal bias is shifted in the direction of the new prior. (E) Single-trial comparison of the Bayesian direction estimate and the PV decoder estimate across stimulus direction. The color codes the stimulus ITD. (F – J) As in (A – E), for a new prior shifted to positive directions. (K – O) As in (A – E), for a new wider prior. (P – T) As in (A – E), for a new narrower prior.

## Impact of the maximum firing rate on the population vector

The binaural correlation (BC) influences both the standard deviation of the noise that corrupts ITD and the maximum firing rates of neurons in the model. Here we include simulations with a constant maximum firing rate to show that the maximum firing rate does not represent uncertainty in the non-uniform population code.

We first repeated the analysis shown in Fig 5, but the maximum firing rate was held constant at 6.4 spikes per stimulus, corresponding to maximum firing rate when BC = 1 (Fig S.2). This is evident in the contrast between the low spike counts seen in Fig 5B and the high spike counts seen in Fig S.2B, where BC = 0.1. The population vector estimate of direction shows increasing bias as BC decreases (Fig S.2C), as in the model where both the width and maximum firing rate change with BC (Fig 5C). This occurs because the maximum firing rate does not influence the shape of the pattern of activity across the population and it is the shape of the pattern of activity that represents the likelihood function. The accuracy of the approximation between the population vector and the Bayesian estimate on single trials is slightly better than in the model where both the width and maximum firing rate change with BC because the constant high firing rate leads to more active neurons (Fig S.2 E and F).

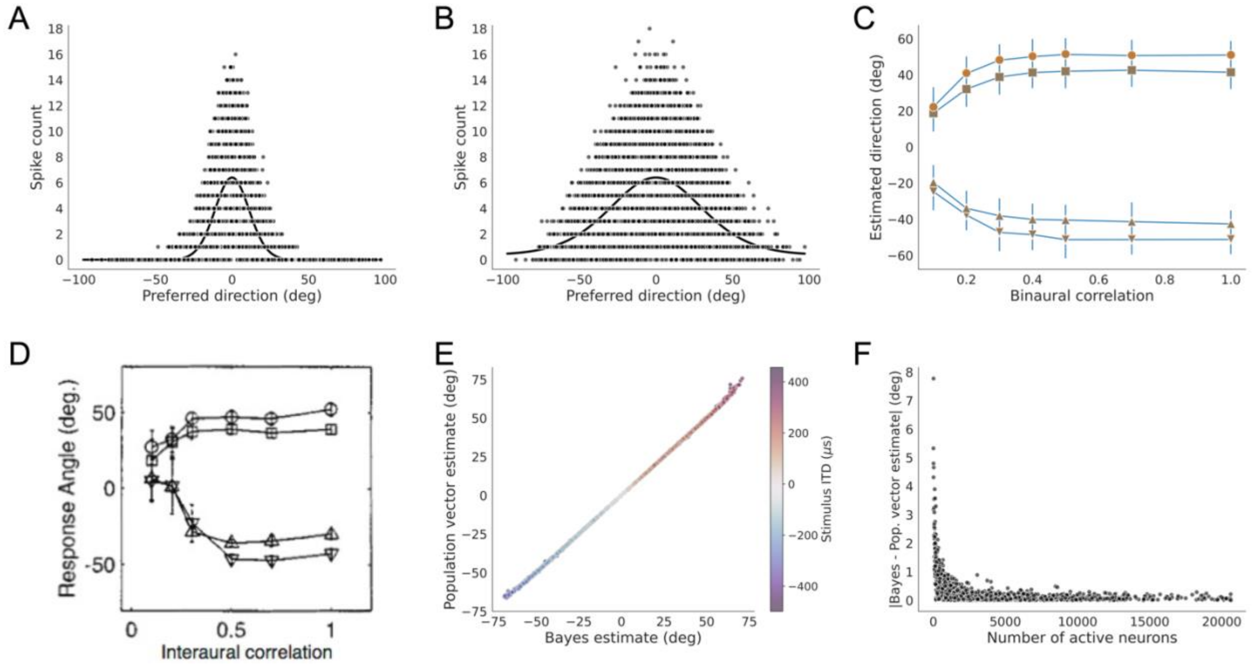


**Fig S.2. Changing binaural correlation with constant high firing rates.** The figure follows Fig. 5 but the maximum firing rate was held constant at the level occurring when BC = 1. (A, B) Example single-trial population responses to stimuli with BC = 1 (A) and BC = 0.1 (B). The solid line is proportional to the likelihood function. (C) Trial averaged comparison of the Bayesian direction estimate (blue) and the population vector decoder estimate (orange) for example stimulus directions of 75 (circles), 55 (squares), -55 (triangles), -75 (inverted triangles) degrees as a function of BC, as in Fischer and Peña (2011) figure 2b. (D) A barn owl's behavior under the same stimulus conditions reproduced from [4]. (E) Single-trial comparison of the Bayesian direction estimate and the population vector decoder estimate combined over stimulus direction and BC. The color represents the stimulus ITD on each trial. (F) Difference between the Bayesian direction estimate and the population vector decoder estimate vs. the number of active neurons combined over stimulus direction and BC. The largest errors occur when no neurons respond to the stimulus in which case the estimate is assumed to be zero.

We also repeated the analysis shown in Fig 5, but with the maximum firing rate held constant at 0.47 spikes per stimulus, corresponding to maximum firing rate when BC = 0.1 (Fig S.3). This is evident in the contrast between the high spike counts seen in Fig 5A and the low spike counts seen in Fig S.3A, where BC = 1. The population vector estimate of direction shows increasing bias as BC decreases (Fig S.3C), as in the model where both the width and maximum firing rate change with BC (Fig 5C). As above, this occurs because the maximum firing rate does not influence the

shape of the pattern of activity across the population and it is the shape of the pattern of activity that represents the likelihood function. However, the accuracy of the approximation between the population vector and the Bayesian estimate on single trials is worse than in the model where both the width and maximum firing rate change with BC because the constant low firing rate leads to fewer active neurons (Fig S.3 E and F), including cases where none of the neurons respond to the stimulus.

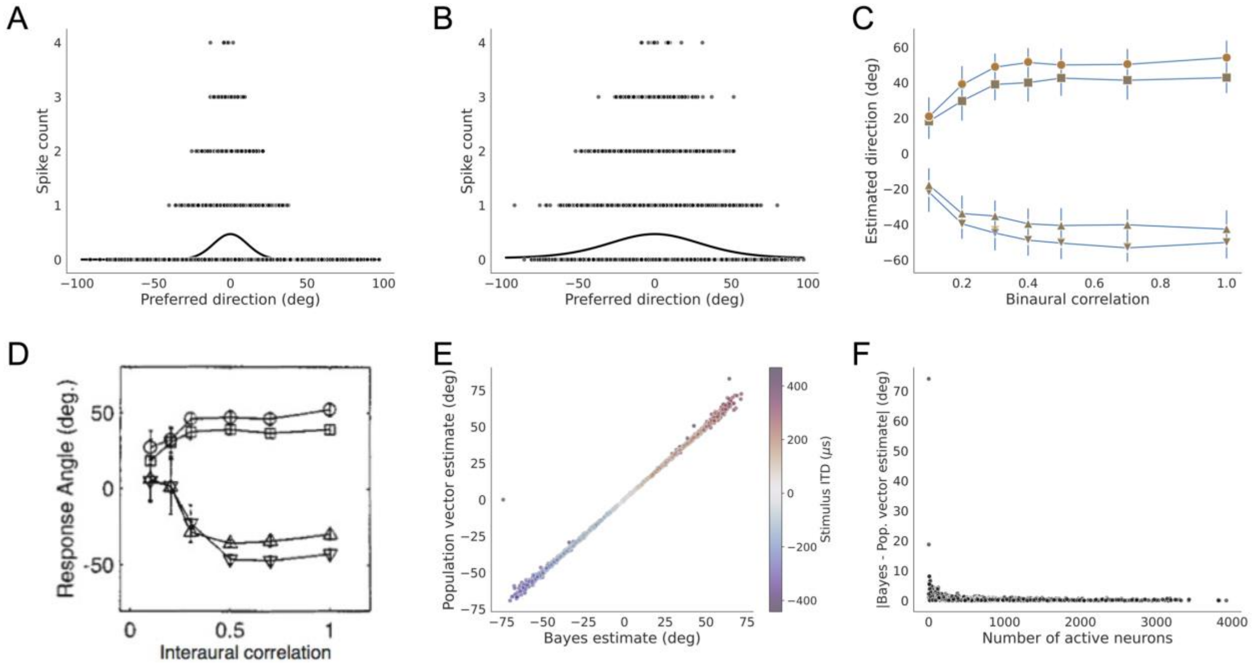


**Fig S.3. Changing binaural correlation with constant low firing rates.** The figure follows Fig. 5 but the maximum firing rate was held constant at the level occurring when BC = 0.1. (A, B) Example single-trial population responses to stimuli with BC = 1 (A) and BC = 0.1 (B). The solid line is proportional to the likelihood function. (C) Trial averaged comparison of the Bayesian direction estimate (blue) and the population vector decoder estimate (orange) for example stimulus directions of 75 (circles), 55 (squares), -55 (triangles), -75 (inverted triangles) degrees as a function of BC, as in Fischer and Peña (2011) figure 2b. (D) A barn owl's behavior under the same stimulus conditions reproduced from [4]. (E) Single-trial comparison of the Bayesian direction estimate and the population vector decoder estimate combined over stimulus direction and BC. The color represents the stimulus ITD on each trial. (F) Difference between the Bayesian direction estimate and the population vector decoder estimate vs. the number of active neurons combined over stimulus direction and BC. The largest errors occur when no neurons respond to the stimulus in which case the estimate is assumed to be zero.

These simulations illustrate that the maximum firing rate does not influence the coding of uncertainty in the model. The bias observed in the population vector estimate as BC decreases is due to the increase in width of population activity. The maximum firing rate only influences the accuracy of the approximation of the Bayesian estimate by the population vector by influencing the number of active neurons on each trial.

# References


1. Fischer BJ, Peña JL. Optimal nonlinear cue integration for sound localization. J Comput Neurosci. 2017;42: 37–52. doi:10.1007/s10827-016-0626-4
2. Fischer BJ. Bayesian estimates from heterogeneous population codes. Neural Networks (IJCNN), The 2010 International Joint Conference on. 2010. pp. 1–7.
3. Fischer BJ, Peña JL. Owl's behavior and neural representation predicted by Bayesian inference. Nat Neurosci. 2011;14: 1061–1066. doi:10.1038/nn.2872
4. Saberi K, Takahashi Y, Konishi M, Albeck Y, Arthur BJ, Farahbod H. Effects of interaural decorrelation on neural and behavioral detection of spatial cues. Neuron. 1998;21: 789–798.
